# Two new species of *Neohelicomyces* (*Tubeufiaceae*, *Tubeufiales*) from southern China revealed by morphological and multi-locus phylogenetic analyses

**DOI:** 10.3897/mycokeys.131.180862

**Published:** 2026-04-21

**Authors:** Li-Juan Zhang, Song Bai, Jian Ma, Yong-Zhong Lu

**Affiliations:** 1 School of Food and Pharmaceutical Engineering, Guizhou Institute of Technology, Guiyang, Guizhou 550003, China Guizhou Academy of Agricultural Sciences Guiyang China; 2 Guizhou Key Laboratory of Agricultural Microbiology, Guizhou Academy of Agricultural Sciences, Guiyang 550009, China Guizhou Institute of Technology Guiyang China; 3 Guizhou Industry Polytechnic College, Guiyang, Guizhou 550008, China Guizhou Industry Polytechnic College Guiyang China

**Keywords:** *
Dothideomycetes
*, helicoid, new taxa, phylogeny, taxonomy

## Abstract

During a survey of helicosporous hyphomycetes in tropical regions of China, four fungal strains were isolated from decaying wood in freshwater habitats of Hainan Province. Multi-locus phylogenetic analyses (LSU, ITS, *tef*1-α, and *rpb*2), coupled with detailed morphological examinations, support the recognition of two new species, *Neohelicomyces baochengensis* and *N. xiangshuiensis*. Comprehensive morphological descriptions, illustrations, taxonomic notes, and phylogenetic evidence are provided to clarify their systematic placement. These findings broaden the current understanding of *Neohelicomyces* diversity and provide further records of the genus from tropical freshwater habitats in Hainan Province.

## Introduction

Helicosporous hyphomycetes are abundant and widely distributed in tropical and subtropical regions, with numerous species recorded from southern China (Morgan 1892; Moore 1957; Rao and Rao 1964; Goos 1985, 1990; Tian et al. 2022; Ma et al. 2024a, 2024b; Lu et al. 2025). Most members of this group are saprobes that colonize dead or decaying plant substrates, particularly lignicolous materials, and inhabit a wide range of ecosystems, including terrestrial, freshwater, marine, and aero-aquatic environments (Lu and Kang 2020; Ma et al. 2023, 2025). Genera such as *Helicoma* Corda, *Helicomyces* Link, *Helicosporium* Nees, *Neohelicomyces* Z.L. Luo, D.J. Bhat & K.D. Hyde, *Neohelicosporium* Y.Z. Lu, J.C. Kang & K.D. Hyde, *Tubeufia* Penz. & Sacc, and *Zalerion* R.T. Moore & Meyers are recognized as valuable fungal resources due to their ability to produce secondary metabolites with novel structures and significant biological activities (Lu et al. 2018a, 2018b; Zeng et al. 2022; Qian et al. 2023; Zhang et al. 2023a, 2023b, 2024; Zheng et al. 2023). These compounds show substantial potential for pharmaceutical development, particularly in antitumor, anticancer, and antibacterial applications (Ma et al. 2024b; Gao et al. 2025; Han et al. 2025; Tan et al. 2025).

*Neohelicomyces* was established by Luo et al. (2017), with *N. aquaticus* designated as the type species based on combined phylogenetic analyses of LSU, ITS, and *tef*1-α sequence data together with morphological evidence. The asexual morph of *Neohelicomyces* is characterized by gregarious colonies; macronematous, mononematous, erect, septate conidiophores; mono- to polyblastic, denticulate, integrated, terminal or intercalary conidiogenous cells; and acropleurogenous or pleurogenous, aseptate or septate, guttulate, hyaline, helicoid conidia (Yang et al. 2023; Ma et al. 2024a, 2024b; Peng et al. 2025). The sexual morph is defined by superficial, solitary to scattered, reddish-brown to brown, subglobose ascomata; 8-spored, bitunicate, cylindric-clavate asci with rounded apices and short pedicels; and multiseriate, narrowly cylindrical, straight to slightly curved, hyaline to pale brown, septate, roughened ascospores (Sun et al. 2025). To date, the genus comprises 36 accepted species (Luo et al. 2017; Lu et al. 2018b; Tibpromma et al. 2018; Crous et al. 2019a, 2019b; Dong et al. 2020; Hsieh et al. 2021; Lin et al. 2025). Species of *Neohelicomyces* have been reported from China, Germany, Italy, Japan, Thailand, the Czech Republic, the Netherlands, and the USA, where they occur as saprobes on a variety of plant substrates (e.g., bamboo and other woody materials) in freshwater and terrestrial habitats (Linder 1929; Goos 1986, 1989; Tsui et al. 2006; Zhao et al. 2007; Ruibal et al. 2009).

In this study, four helicosporous hyphomycete isolates representing two distinct taxonomic lineages were collected from freshwater habitats in Hainan Province, China. Detailed morphological observations, illustrations, and multigene phylogenetic analyses were undertaken to characterize these taxa. Based on this integrative evidence, two previously undescribed species, *Neohelicomyces baochengensis* and *N. xiangshuiensis*, are formally proposed herein. These findings expand our understanding of *Neohelicomyces* and provide new insights into its diversity in tropical freshwater habitats.

## Materials and methods

### Sample collection, specimen examination, and isolation

Decaying wood pieces submerged in water were collected from Baoting Li and Miao Autonomous County, Hainan Province, China. Samples were taken to the laboratory in plastic bags with the collection details, including localities and dates (Rathnayaka et al. 2024). The microscopic features were examined and photographed using a stereomicroscope (SMZ-168, Nikon, Japan) and an ECLIPSE Ni compound microscope (Nikon, Tokyo, Japan) with a Canon 90D digital camera (Canon, China). Measurements were made using Tarosoft Image Frame Work software. Photo plates were made using Adobe Photoshop CC 2019 (Adobe Systems, USA).

Single-spore isolation was carried out according to the methods described by Senanayake et al. (2020), and the germinated conidia were aseptically transferred to fresh PDA plates. Morphological characters of fungal colonies, including color, shape, and size, were documented. Dried specimens were deposited in the Herbarium of Guizhou Academy of Agricultural Sciences (Herb. GZAAS), Guiyang, China. Pure cultures were deposited in the Guizhou Culture Collection (**GZCC**), Guiyang, China. MycoBank numbers of newly obtained species were registered in the MycoBank database (https://www.mycobank.org/).

### DNA extraction, PCR amplification, and sequencing

Fresh fungal mycelia were scraped from colonies grown on PDA plates and transferred to a 1.5 mL microcentrifuge tube using a sterilized lancet for genomic DNA extraction. Genomic DNA was extracted using the Biospin Fungus Genomic DNA Extraction Kit (BioFlux, China). The primers LR0R/LR5, ITS5/ITS4, EF1-983F/EF1-2218R, and fRPB2-5F/fRPB2-7cR were employed to amplify the large ribosomal subunit (LSU; Vilgalys and Hester 1990), internal transcribed spacer (ITS; White et al. 1990), translation elongation factor 1-alpha (*tef*1-α; Rehner and Buckley 2005), and RNA polymerase II second largest subunit (*rpb*2; Liu et al. 1999) sequence fragments, respectively. Polymerase chain reaction (PCR) was performed in a 50 μL reaction mixture containing 2 μL DNA, 2 μL of each forward and reverse primer, and 44 μL of 1.1× T3 Super PCR Mix (including 17 μL distilled-deionized water; Tsingke Biotech, Chongqing, China). The PCR conditions were as reported by Ma et al. (2023). The PCR products were purified and sequenced with the same primers at Beijing Tsingke Biotechnology Co., Ltd.

### Phylogenetic analyses

Newly obtained sequences were checked and assembled using BioEdit v.7.0.5.3 (Hall 1999) and SeqMan v.7.0.0 (DNASTAR, Madison, WI, USA; Swindell and Plasterer 1997), respectively. The taxa used in this study were selected based on the closest matches from BLASTn search results (Table 1; https://www.ncbi.nlm.nih.gov/) and from previous studies. Multiple sequences were aligned using MAFFT v.7.473 (https://mafft.cbrc.jp/alignment/server/; Katoh et al. 2019). The dataset was trimmed using trimAl v.1.2rev59 software (Capella-Gutiérrez et al. 2009). A combined sequence dataset was created using SequenceMatrix-Windows 1.7.8 software (Vaidya et al. 2011).

**Table 1.** Taxa used in this study and their GenBank accession numbers.

Taxon	Strain	GenBank accessions			
		LSU	ITS	*tef*1-α	*rpb*2
*Helicotubeufia hydei*	MFLUCC 17-1980^T^	MH290026	MH290021	MH290031	MH290036
*Helicotubeufia jonesii*	MFLUCC 17-0043^T^	MH290025	MH290020	MH290030	MH290035
*Muripulchra aquatica*	KUMCC 15-0276	KY320551	KY320534	KY320564	MH551058
*Muripulchra aquatica*	MFLUCC 15-0249^T^	KY320549	KY320532	N/A	N/A
*Neohelicomyces acropleurogenus*	CGMCC 3.25549^T^	PP639450	PP626594	PP596351	PP596478
*Neohelicomyces aquaticus*	MFLUCC 16-0993^T^	KY320545	KY320528	KY320561	MH551066
*Neohelicomyces aquisubtropicus*	GZCC 23-0080^T^	PQ098537	PQ098499	PV768327	PV768336
*Neohelicomyces aseptatus*	CGMCC 3.25564^T^	PP639451	PP626595	PP596352	PP596479
*Neohelicomyces astrictus*	HKAS 105122^T^	PQ898796	PQ898760	PV040811	N/A
***Neohelicomyces baochengensis***	**GZCC 25-0669^T^**	**PX848711**	**PX848697**	**PZ095451**	**PZ095447**
***Neohelicomyces baochengensis***	**GZCC 25-0670**	**PX848712**	**PX848698**	**PZ095452**	**PZ095448**
*Neohelicomyces brunneus*	HKAS 105147^T^	PQ898805	PQ898768	PV040818	N/A
*Neohelicomyces coffeae*	GMBCC 2225^T^	PX308848	PX308843	PX314510	PX314514
*Neohelicomyces dehongensis*	MFLUCC 18-1029^T^	MN913709	NR_171880	MT954393	N/A
*Neohelicomyces denticulatus*	GZCC 19-0444^T^	MW133855	OP377832	N/A	N/A
*Neohelicomyces deschampsiae*	CPC 33686^T^	MK442538	MK442602	N/A	N/A
*Neohelicomyces edgeworthiae*	CGMCC 3.25565^T^	PP639453	PP626597	PP596354	PP596481
*Neohelicomyces grandisporus*	KUMCC 15-0470^T^	KX454174	KX454173	N/A	MH551067
*Neohelicomyces guizhouensis*	GZCC 23-0725^T^	PP512973	PP512969	PP526727	PP526733
*Neohelicomyces guttulatus*	CGMCC 3.25550^T^	PP639454	PP626598	PP596355	N/A
*Neohelicomyces hainanensis*	GZCC 22-2009^T^	OP508774	OP508734	OP698085	OP698074
*Neohelicomyces helicosporus*	GZCC 23-0633^T^	PP512975	PP512971	PP526729	PP526735
*Neohelicomyces hyalosporus*	GZCC 16-0086^T^	MH558870	MH558745	MH550936	MH551064
*Neohelicomyces hydei*	GZCC 23-0727^T^	PP512977	N/A	PP526731	PP526737
*Neohelicomyces lignicola*	CGMCC 3.25551^T^	PP639456	PP626600	PP596357	PP596483
*Neohelicomyces longisetosus*	NCYU-106H1-1-1^T^	N/A	MT939303	N/A	N/A
*Neohelicomyces macrosporus*	CGMCC 3.25552^T^	PP639457	PP626601	PP596358	PP596484
*Neohelicomyces maolanensis*	GZCC 23-0079^T^	PQ098529	N/A	PQ490683	PQ490677
*Neohelicomyces melaleucae*	CPC 38042^T^	MN567661	MN562154	MN556835	N/A
*Neohelicomyces pallidus*	CBS 271.52	AY856887	AY916461	N/A	N/A
*Neohelicomyces pallidus*	CBS 962.69	AY856886	AY916460	N/A	N/A
*Neohelicomyces pandanicola*	KUMCC 16-0143^T^	MH260307	MH275073	MH412779	N/A
*Neohelicomyces puerensis*	GMBCC 2217^T^	PX308846	PQ737369	PX314508	PX314512
*Neohelicomyces qixingyaensis*	CGMCC 3.25569^T^	PP639458	PP626602	PP596359	PP596485
*Neohelicomyces saprobicus*	GZCC 23-0743^T^	PX625152	PX625156	PX830980	N/A
*Neohelicomyces sexualis*	HGUP 24-0021^T^	PQ570861	PQ570844	N/A	N/A
*Neohelicomyces submersus*	MFLUCC 16-1106^T^	KY320547	KY320530	N/A	MH551068
*Neohelicomyces subtropicus*	GZCC 23-0076^T^	PQ098530	PQ098492	PQ490685	PQ490679
*Neohelicomyces terrestris*	GZCC 23-0399^T^	PX575662	PX575639	PX512845	PX512836
*Neohelicomyces thailandicus*	MFLUCC 11-0005^T^	MN913696	NR_171882	N/A	N/A
*Neohelicomyces tropicus*	GZCC 25-0661^T^	PX575664	PX575641	PX512847	PX512838
*Neohelicomyces uniramulosus*	GZCC 25-0750^T^	PX625154	PX625158	N/A	N/A
*Neohelicomyces wuzhishanensis*	GZCC 23-0410^T^	PQ098532	PQ098494	PV768325	PV768334
***Neohelicomyces xiangshuiensis***	**GZCC 25-0671^T^**	**N/A**	**PX848699**	**PZ095453**	**PZ095449**
***Neohelicomyces xiangshuiensis***	**GZCC 25-0672**	**N/A**	**PX848700**	**PZ095454**	**PZ095450**
*Neohelicomyces xiayadongensis*	CGMCC 3.25568^T^	PP639460	PP626604	PP596361	PP596487
*Neohelicomyces yunnanensis*	GZCC 23-0735^T^	PP664113	PP664109	N/A	N/A
*Tubeufia guttulata*	GZCC 23-0404^T^	OR030834	OR030841	OR046678	OR046684
*Tubeufia hainanensis*	GZCC 22-2015^T^	OR030835	OR030842	OR046679	OR046685
*Tubeufia javanica*	MFLUCC 12-0545^T^	KJ880036	KJ880034	KJ880037	N/A
*Tubeufia krabiensis*	MFLUCC 16-0228^T^	MH558917	MH558792	MH550985	MH551118
*Tubeufia latispora*	MFLUCC 16-0027^T^	KY092412	KY092417	KY117033	MH551119
*Tubeufia laxispora*	MFLUCC 16-0232^T^	KY092408	KY092413	KY117029	MF535287
*Tubeufia mackenziei*	MFLUCC 16-0222^T^	KY092410	KY092415	KY117031	MF535288
*Tubeufia muriformis*	GZCC 22-2039^T^	OR030836	OR030843	OR046680	OR046686
*Tubeufia nigroseptum*	CGMCC 3.20430^T^	MZ853187	MZ092716	OM022002	OM022001
*Tubeufia pandanicola*	MFLUCC 16-0321^T^	MH260325	MH275091	N/A	N/A

**Notes**. Newly generated sequences are shown in bold black. “^T^” denotes ex-type strains. “N/A” indicates data that are unavailable in GenBank.

Maximum likelihood (ML) analysis was performed using the RAxML-HPC v.8 on XSEDE (8.2.12) tool, with a GTRGAMMA approximation and rapid bootstrap analysis followed by 1,000 bootstrap replicates (Stamatakis 2014). Bayesian inference (BI) analysis was performed using MrBayes (3.2.7a) on XSEDE via CIPRES (Stamatakis 2014). The aligned FASTA file was converted to a Nexus format file using AliView (Daniel et al. 2010). The best-fit evolutionary model for each locus was determined using MrModeltest v.2.3.10 (Nylander et al. 2008). The GTR+G+I substitution model was selected for LSU, ITS, and *tef*1-α, whereas the SYM+I+G model was applied to *rpb*2. The Bayesian posterior probabilities (PP) were determined based on Bayesian Markov chain Monte Carlo (BMCMC) sampling (Huelsenbeck and Ronquist 2001). Four simultaneous Markov chains were run for 10,000,000 generations, and trees were sampled every 1,000 generations. The burn-in phase was set to 25%, and the remaining trees were used to calculate posterior probabilities (PP).

Phylogenetic trees were edited using FigTree v.1.4.4 and Adobe Illustrator CC 2019 (v.23.1.0; Adobe Systems, USA).

### Phylogenetic analysis results

The phylogenetic positions of the four novel strains were determined using a multi-locus phylogenetic approach. The concatenated sequence matrix comprised 3,413 characters (LSU: 1–857, ITS: 858–1,430, *tef*1-α: 1,431–2,342, and *rpb*2: 2,343–3,413) across 57 taxa. Base frequencies and rates were *A* = 0.249478, *C* = 0.246310, *G* = 0.255991, and *T* = 0.248220; substitution rates were AC = 1.227845, AG = 5.983718, AT = 2.837414, CG = 1.087601, CT = 9.238154, and GT = 1.000000. The distribution shape parameter *α* equaled 0.177658.

**Figure 1.**
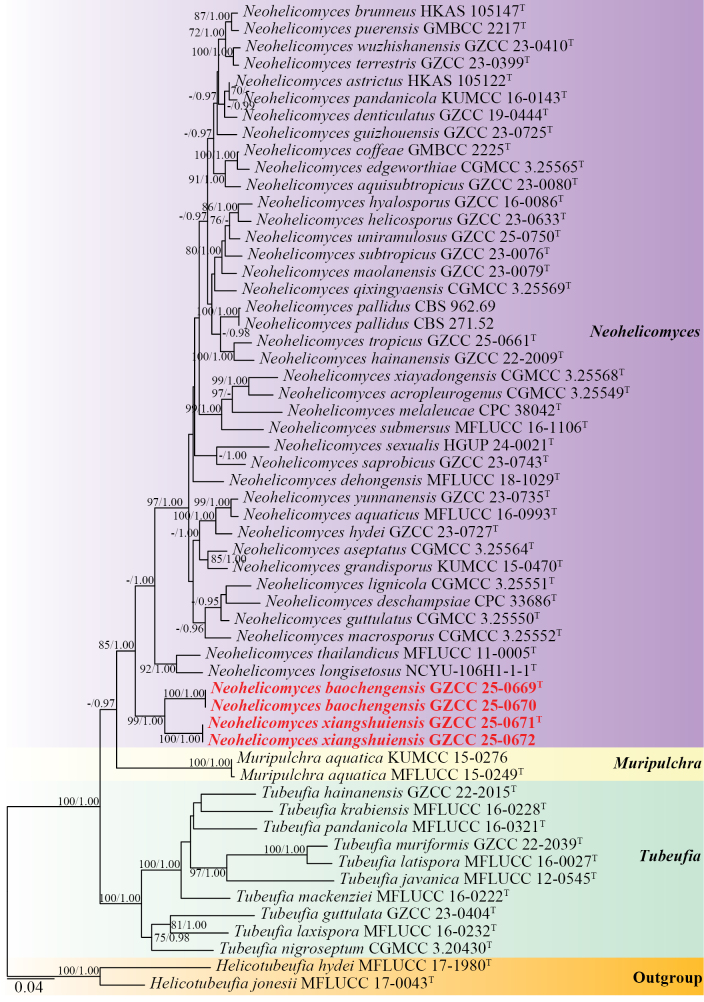
Phylogenetic tree generated from maximum likelihood (ML) analysis based on the combined LSU, ITS, *tef*1-α, and *rpb*2 sequence data. ML bootstrap support (≥ 70%) and Bayesian posterior probabilities (≥ 0.95) are indicated near their respective nodes. Both ML and BI analyses produced congruent topologies. Hyphen (“-”) indicates a value lower than 70% for ML and a posterior probability lower than 0.95 for Bayesian inference. The tree is rooted with *Helicotubeufia hydei* (MFLUCC 17-1980) and *H. jonesii* (MFLUCC 17-0043). Ex-type strains are denoted with “^T,^” and newly obtained strains are in bold red.

The multigene phylogenetic tree (Fig. 1) indicates that the four new collections represent two distinct species within *Neohelicomyces*. The newly obtained isolates GZCC 25-0669 and GZCC 25-0670 form a well-supported sister clade to GZCC 25-0671 and GZCC 25-0672, with 99% ML and 1.00 PP support.

## Taxonomy

### Neohelicomyces baochengensis

Taxon classificationFungiTubeufialesTubeufiaceae

L.J. Zhang, J. Ma & Y.Z. Lu sp. nov.

444631D5-10AB-505F-89FC-5790673E39A2

905188

Fig. 2

#### Etymology.

The species epithet “*baochengensis*’’ refers to the type locality, “Baocheng Town, Baoting Li and Miao Autonomous County, China.”

#### Holotype.

GZAAS 25-0697.

#### Description.

***Saprobic*** on decaying wood in freshwater habitats. **Sexual morph**: Undetermined. **Asexual morph**: Hyphomycetous, helicosporous. ***Colonies*** on natural substrate superficial, effuse, gregarious, with masses of crowded, glistening conidia, white to brown. ***Mycelium*** partly immersed, partly superficial, composed of hyaline to pale brown, branched, septate, smooth hyphae. ***Conidiophores*** 125–185 × 4–5 μm (x̄ = 149 × 4.6 μm, *n* = 20), macronematous, mononematous, erect, cylindrical, flexuous, widest at the base, tapering towards a narrow apex, branched or unbranched, septate, brown at base, subhyaline to pale brown towards apex, thick-walled. ***Conidiogenous cells*** 8.5–15 × 2.5–4 μm (x̄ = 11 × 3.3 μm, *n* = 25), holoblastic, monoblastic, or polyblastic, integrated, terminal, or intercalary, cylindrical, with bladder-like protrusions, 2.5–6.5 × 1.5–2.5 μm, pale brown to brown, smooth-walled. ***Conidia*** solitary, acropleurogenous, helicoid, developing on tooth-like or bladder-like protrusions, 17.5–21.5 μm diam. and conidial filament 2.5–3 μm wide (x̄ = 19 × 2.7 μm, *n* = 20), 120–147 μm long (x̄ = 130 μm, *n* = 30), tightly coiled up to 2^1^/_2_ times, becoming loosely coiled in water, septate, guttulate, hyaline, smooth-walled.

**Figure 2.**
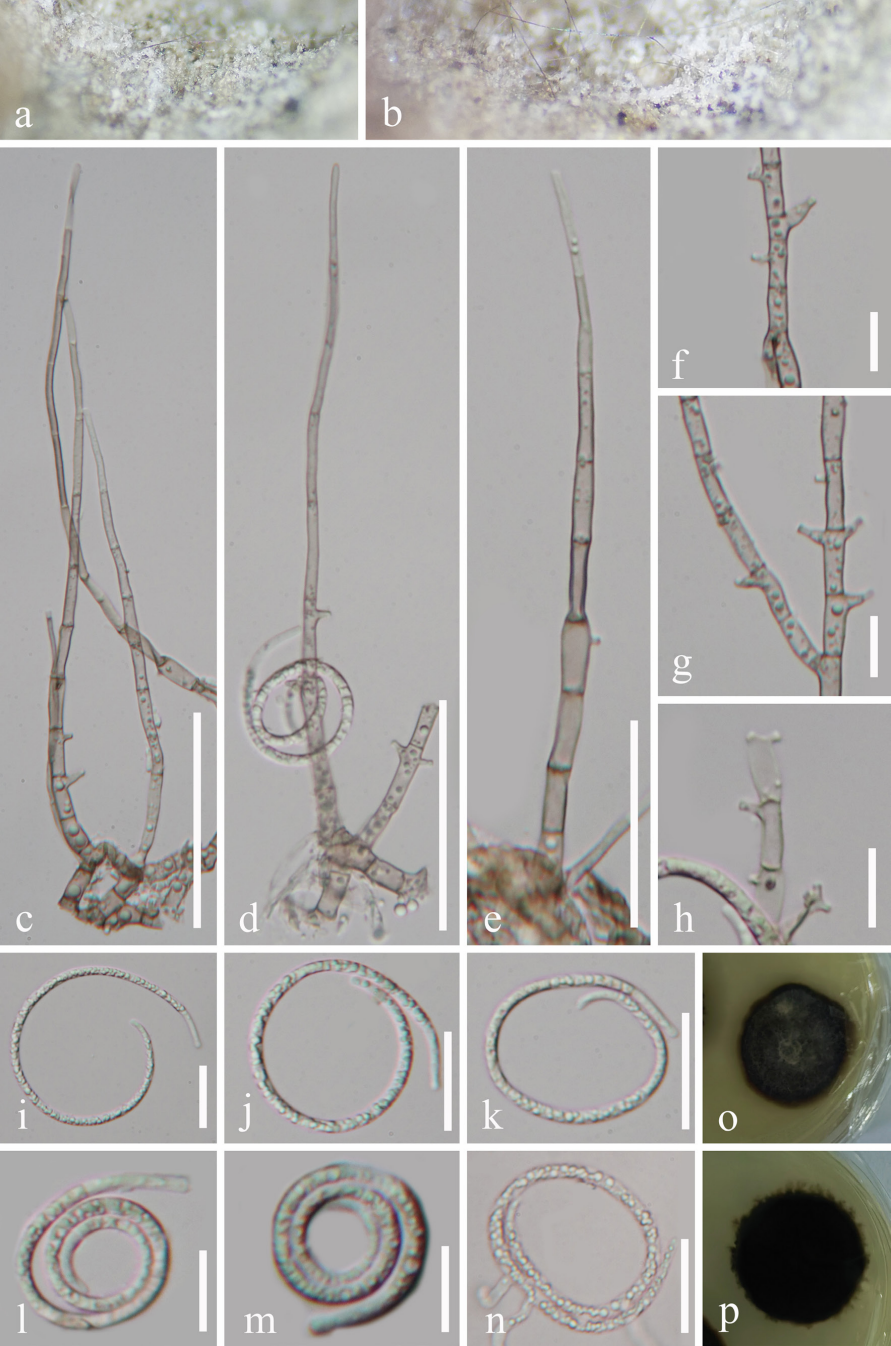
*Neohelicomyces baochengensis* (GZAAS 25-0697, holotype). **a, b**. Colonies on the host surface; **c–e**. Conidiophores and conidiogenous cells; **f–h**. Conidiogenous cells; **i–m**. Conidia; **n**. Germinated conidium; **o, p**. Colonies on PDA from above and below. Scale bars: 50 μm (**c**); 40 μm (**d**); 30 μm (**e**); 20 μm (**i–k, n**); 10 μm (**f–h, l, m**).

#### Culture characteristics.

Conidia germinated on PDA and produced germ tubes within 13 h. Colonies on PDA reached 29 mm in diameter after 42 days of incubation at 25 °C, with a circular shape, raised surface, entire margin, and brown to black coloration.

#### Material examined.

China • Hainan Province, Baoting Li and Miao Autonomous County, Baocheng Town, on decaying wood in a freshwater habitat, 18 March 2025, Li-Juan Zhang & Jian Ma, BT80 (GZAAS 25-0697, holotype), ex-type living culture GZCC 25-0669; *ibid*., BT81 (GZAAS 25-0698, paratype), living culture GZCC 25-0670.

#### Notes.

Morphologically, *Neohelicomyces baochengensis* (GZAAS 25-0697) resembles *N. guizhouensis* (HKAS 134924) in having macronematous, mononematous, erect, flexuous, cylindrical, branched, septate conidiophores; holoblastic, monoblastic, or polyblastic, integrated, cylindrical conidiogenous cells; and hyaline, guttulate, septate, helicoid conidia (Ma et al. 2024b). However, *N. baochengensis* (GZAAS 25-0697) differs from *N. guizhouensis* (HKAS 134924) by its shorter conidiophores (125–185 μm vs. up to 288 μm) and fewer coilings (up to 2^1^/_2_ times *vs*. 2^3^/_4_–3^1^/_2_ times) (Ma et al. 2024b). Molecular phylogenetic analyses showed that *N. baochengensis* and *N. xiangshuiensis* form a sister clade, which is clearly distinct from *N. guizhouensis* (Fig. 1). Therefore, based on morphological comparison and multigene phylogenetic analyses, we describe GZCC 25-0669 and GZCC 25-0670 as a new species, *N. baochengensis*.

### Neohelicomyces xiangshuiensis

Taxon classificationFungiTubeufialesTubeufiaceae

L.J. Zhang, J. Ma & Y.Z. Lu sp. nov.

2DF14795-8F5A-52B3-A4FA-D419608B9A62

905189

Fig. 3

#### Etymology.

The species epithet “*xiangshuiensis*’’ refers to the type locality, “Xiangshui Town, Baoting Li and Miao Autonomous County, China.”

#### Holotype.

GZAAS 25-0699.

#### Description.

***Saprobic*** on decaying wood in freshwater habitats. **Sexual morph**: Undetermined. **Asexual morph**: Hyphomycetous, helicosporous. ***Colonies*** on natural substrate superficial, gregarious, with little crowded, glistening conidia, white. ***Mycelium*** partly immersed, partly superficial, composed of hyaline to pale brown, branched, septate, smooth hyphae. ***Conidiophores*** 147–259 × 3.8–5.7 μm (x̄ = 194.5 × 4.9 μm, *n* = 25), macronematous, mononematous, erect, cylindrical, straight, or slightly flexuous, branched, or unbranched, septate, subhyaline to brown, thick-walled. ***Conidiogenous cells*** 9–15.5 × 3–4.5 μm (x̄ = 13 × 3.7 μm, *n* = 25), holoblastic, monoblastic, or polyblastic, integrated, terminal, or intercalary, cylindrical, with denticles, subhyaline to brown, smooth-walled. ***Conidia*** solitary, acropleurogenous, helicoid, tapering towards the rounded ends, developing on a tooth-like protrusion, 15–17 μm diam. and conidial filament 2–3 μm wide (x̄ = 16 × 2.5 μm, *n* = 20), 92.5–126 μm long (x̄ = 109.5 μm, *n* = 20), tightly coiled up to 3^3^/_4_ times, becoming loosely coiled when the conidia are young and not becoming loose when mature in water, septate, guttulate, hyaline, smooth-walled.

**Figure 3.**
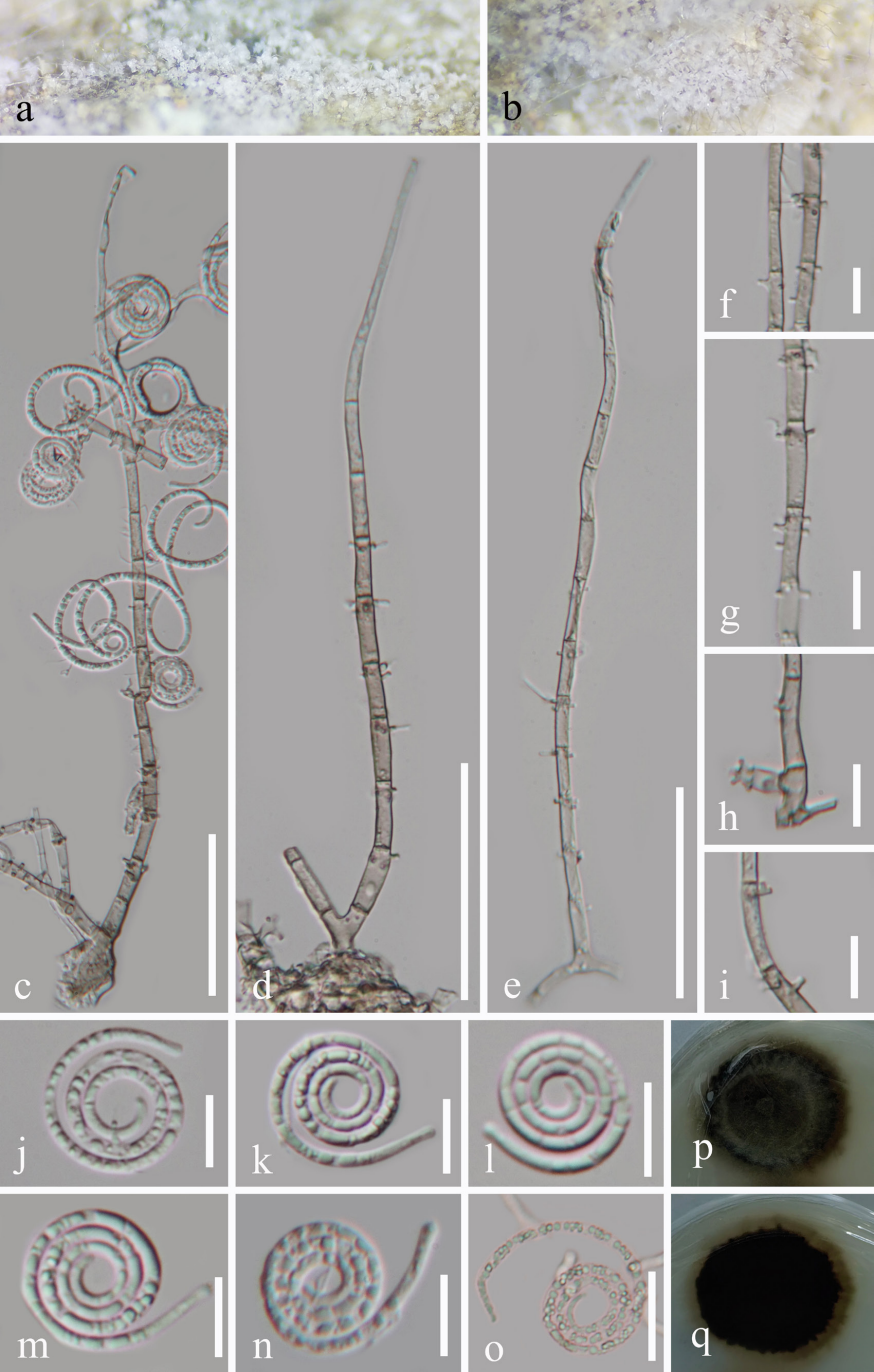
*Neohelicomyces xiangshuiensis* (GZAAS 25-0699, holotype). **a, b**. Colonies on the host surface; **c–e**. Conidiophores, conidiogenous cells, and conidia; **f–i**. Conidiogenous cells; **o**. Germinated conidium; **j–n**. Conidia; **p, q**. Colonies on PDA from above and below. Scale bars: 50 μm (**c–e**); 20 μm (**o**); 10 μm (**f–n**).

#### Culture characteristics.

Conidia germinated on PDA and produced germ tubes within 15 h. Colonies on PDA reached 36 mm in diameter after 45 days of incubation at 25 °C with an irregular shape, flat surface, undulate margin, pale brown to brown; the reverse was brown to black.

#### Material examined.

China • Hainan Province, Baoting Li and Miao Autonomous County, Baocheng Town, on decaying wood in a freshwater habitat, 18 March 2025, Li-Juan Zhang & Jian Ma, BT8A (GZAAS 25-0699, holotype), ex-type living culture GZCC 25-0671; *ibid*., BT8B (GZAAS 25-0700, paratype), living culture GZCC 25-0672.

#### Notes.

In the phylogenetic analyses (Fig. 1), *Neohelicomyces xiangshuiensis* (GZCC 25-0671 and GZCC 25-0672) formed a sister lineage to *N. baochengensis* (GZCC 25-0669 and GZCC 25-0670) with 99% ML and 1.00 PP statistical support. Based on the molecular sequence comparison, our isolate (GZCC 25-0671, ex-type) differs from *N. baochengensis* (GZCC 25-0669, ex-type) by 82/896 bp for ITS (9.2%, gaps 36 bp), 30/931 bp (3.2%, without gaps) for *tef*1-α, and 63/1081 bp (5.8%, without gaps) for *rpb*2. Morphologically, *N. xiangshuiensis* (GZAAS 25-0699) can be distinguished from *N. baochengensis* (GZAAS 25-0697) by its smaller conidia (15–17 μm vs. 17.5–21.5 μm in diameter and 92.5–126 μm vs. 120–147 μm in length). Therefore, *Neohelicomyces xiangshuiensis* is proposed here as a new species based on multigene phylogenetic analyses and distinctive morphological characteristics.

## Discussion

Currently, the genus *Neohelicomyces* comprises 38 species, including the two newly described taxa, *N. baochengensis* and *N. xiangshuiensis*. Morphologically, *Neohelicomyces* and *Neohelicosporium* are extremely similar, both characterized by macronematous, mononematous, erect, cylindrical, straight or slightly flexuous, septate conidiophores; mono- to polyblastic, denticulate, cylindrical, integrated conidiogenous cells that are terminal or intercalary; and acropleurogenous or pleurogenous, aseptate or septate, guttulate, hyaline, helicoid conidia (Kuo and Goh 2018a, 2018b, 2021; Dayarathne et al. 2019; Jayasiri et al. 2019; Xiao et al. 2023). However, *Neohelicomyces* can be readily differentiated from *Neohelicosporium* by its shorter conidiophores that are rarely branched (Linder 1929; Goos 1986, 1989; Tsui et al. 2006; Zhao et al. 2007; Lu et al. 2017, 2022, 2023a, 2023b; Zhao et al. 2026).

The phylogenetic analyses indicate that the newly described species, *N. baochengensis* and *N. xiangshuiensis*, form a well-supported clade basal to *Neohelicomyces* species (85% ML/1.00 PP, Fig. 1). According to a BLASTn search on NCBI GenBank, the ITS, LSU, *tef*1-α, and *rpb*2 sequences of the new isolate (*Neohelicomyces baochengensis*) share 85.88% similarity with *N. thailandicus*, 99.4% similarity with *Muripulchra aquatica*, 95.36% similarity with *N. coffeae*, and 89.36% similarity across 84% of the query sequence coverage with *N. grandisporus*, respectively. Morphologically, *N. baochengensis* and *N. xiangshuiensis* conform to the diagnostic features of the genus *Neohelicomyces* (Lu et al. 2018b; Ma et al. 2024b). To accurately identify the two newly isolated species, we conducted genus-level analyses using single-gene and multigene datasets, as well as molecular data for *Tubeufiaceae* referenced from Ma et al. (2024b).

The discovery of these two new species expands the known diversity of *Neohelicomyces* and enhances understanding of phylogenetic relationships within helicosporous hyphomycetes of *Tubeufiaceae*. Furthermore, the close morphological resemblance among species in this group underscores the limitations of morphology-based identification. These findings indicate that reliable delimitation of helicosporous taxa requires an integrative approach that combines detailed morphological observations with multi-locus phylogenetic analyses.

## Supplementary Material

XML Treatment for Neohelicomyces baochengensis

XML Treatment for Neohelicomyces xiangshuiensis

## Additional information

### Use of AI

No use of AI was reported.

### Author ORCIDs

Li-Juan Zhang https://orcid.org/0000-0002-3234-6757

Song Bai https://orcid.org/0000-0002-1972-2834

Jian Ma https://orcid.org/0009-0008-1291-640X

Yong-Zhong Lu https://orcid.org/0000-0002-1033-5782

### Data availability

All of the data that support the findings of this study are available in the main text.
